# The Relative Roles of Peer and Parent Predictors in Minor Adolescent Delinquency: Exploring Gender and Adolescent Phase Differences

**DOI:** 10.3389/fpubh.2018.00242

**Published:** 2018-09-13

**Authors:** Ivy N. Defoe, Judith Semon Dubas, Marcel A. G. van Aken

**Affiliations:** ^1^Annenberg Public Policy Center, University of Pennsylvania, Philadelphia, PA, United States; ^2^Department of Developmental Psychology, Faculty of Social Sciences, Utrecht University, Utrecht, Netherlands

**Keywords:** delinquency, peer pressure, peer norms, adolescence, parenting, adolescent phase, gender

## Abstract

Social learning theories assume that delinquent peer norms and/or peer pressure are the components of delinquent peer socialization that lead to subsequent adolescent delinquency. However, these specific peer influences are rarely investigated. Moreover, social learning theories such as coercion theory posit that parenting behaviors also play an important role in the development or prevention of delinquency. However, surprisingly, little research has investigated whether parent behaviors could moderate the link between the above-described peer influences and adolescent delinquency. Hence, using structural equation modeling, the current 1-year longitudinal study investigated these questions among ethnically-diverse Dutch adolescents (*N* = 602; *M*_age_ = 13.50; 46.42% female at baseline), who were mostly between12 and 15 years old. Additionally, using multi-group models, and a stringent *p*-value of *p* < 0.01, we explored whether gender and adolescent phase (i.e., early versus middle adolescence) further moderated these links. The majority of the analyses, resulted in non-significant findings. Specifically, in our non-multi group model, we found no significant peer, and family effects for the entire sample. However, for our multi-group models, we found that higher levels of negative mother-adolescent relationship quality exacerbated the link between peer pressure and subsequent early adolescent boys' delinquency 1 year later, while low levels of mother-adolescent negative relationship quality reversed the association. That is, low levels of mother-adolescent negative relationship quality attenuated the link from higher levels of peer pressure to higher levels of delinquency, but only in early adolescent boys. These findings existed above and beyond significant links from prior adolescent delinquency (T1) to future adolescent delinquency (T2). To conclude, although this was not the case for most adolescents, for *early adolescent boys* fewer negative interactions between mother and adolescents at an earlier time point (in advance) could potentially curtail the negative effects that delinquent peer pressure has on delinquency in the future. Implications for theory and practice are discussed.

## Introduction

Delinquency concerns a variety of externalizing behaviors that violate legal and social rules at a high personal and societal cost. In the current study, we investigate social precursors of delinquent behaviors such as stealing, purchasing stolen goods and vandalism. It has been consistently documented that these type of minor delinquent behaviors show accelerated growth or peaks during adolescence [e.g., (1)], and that delinquent peer affiliation is one of the strongest predictors of juvenile delinquency (2). Criminological variants of social learning theories [e.g., (3–5) that posit that *modeling* links delinquent peer affiliation to subsequent adolescent delinquency, are among the leading theories of peer similarity in delinquency. It is often assumed that modeling is facilitated via conformity to perceived delinquent peer norms (i.e., indirect peer pressure) or via direct/overt peer pressure (6). However, these specific peer affiliation mechanisms in relation to adolescent delinquency are less frequently investigated compared to whether having delinquent peers (i.e., mere delinquent peer affiliation) predicts adolescent delinquency. Although such peer influences become increasingly strong during adolescence (7), a meta-analysis showed that parent-adolescent relationship quality is also an important predictor of adolescent delinquency (8). A critically valid—yet understudied-follow-up question is whether having poor relationships with parents make adolescents more susceptible to adverse peer effects on adolescent delinquency. That is, an abundance of studies exists that investigated these peer and parent predictors independently, but there are fewer studies on possible interactions between these two types of social influences. Accordingly, the primary goal of the current longitudinal study is to investigate whether negative mother-adolescent relationship quality moderates the hypothesized links between delinquent peer norms and perceived peer pressure to engage in delinquency and subsequent delinquency in adolescent boys and girls.

As individuals transition from childhood to adolescence they gradually receive more independence from parents, and the subsequently begin to spend increasingly more time with peers. Thus in that sense, peer influence is ubiquitous during adolescence. Peer influence during adolescence takes place via *peer socialization or peer affiliation*, which consists of an adolescent accepting or changing his or her behavior due to perceived peer norms or peer pressure (9). Peer (social) norms is defined as perceived (thus perhaps not actual) attitudes, behaviors, and beliefs that are regarded as acceptable within a peer group, whereas (overt/perceived) peer pressure is defined as direct pressure exerted on an individual to conform to a particular peer group behavior (9). Peer pressure might be a method to enforce peer group norms (10, 11), however, peer norm internalization can manifest itself without the presence of overt/direct pressure from peers. That is, individuals might still feel indirect pressure to conform to peer norms, without peers pressuring them to engage in a particular behavior. In fact, empirical work suggests that the influence of peers on externalizing problems might manifest primarily—but not exclusively—via indirect pressure to conform to peer norms rather than via direct overt peer pressure (9). However, most of such empirical evidence comes from studies focusing on adolescent substance use [see e.g., (12)], whereas studies on adolescent delinquency (e.g., theft) are lacking.

The Coercion theory is a prominent developmental theory that takes both peer and parent influences on youth externalizing problems development into account (13, 14). However, this theory posits that predictors of delinquency development typically first emerge at home, as the home environment is the first socializing context for children [(13, 14); see also (5)]. It can be extrapolated from this theory that parent-adolescent negative interactions, teach adolescents to behave in a deviant manner, which attracts them to delinquent peers. In other words, parent-adolescent negative interactions could increase the likelihood that adolescents affiliate with—and become more influenced by—delinquent peers. Such delinquent peer affiliation is known to subsequently foster delinquency development in adolescents [(13, 14); see also (5)]. Although according to coercion theory, poor parent-child relationship quality instigates the process of delinquency development, during adolescence, however, deviant peer affiliations become a stronger predictor of adolescent delinquency [e.g., (2)]. This is perhaps not surprising, as individuals tend to gravitate more toward their peers (compared to their parents) during adolescence. Nevertheless, there is some empirical evidence showing that parents can still play a role in adolescents' behavior even when accounting for similar peer (and sibling) influences (15–19). For example, a recent 4-year longitudinal cross-lagged panel study demonstrated that parent-adolescent negative interactions—but not parent externalizing problems—predicted adolescent delinquency and aggression above and beyond significant effects of friends' and siblings' externalizing problem behavior (15). Defoe et al. (15) concluded that parents and friends might play a differential role in adolescent delinquency, that is, whereas the delinquent behavior of friends determines the kinds of delinquent behaviors in which adolescents engage, it is the relationship quality between parents (particularly mothers) and their adolescent offspring that predict whether the adolescent gets involved in delinquency.

Thus, some empirical evidence exists for independent and unique effects of parent-adolescent relationship quality and peer delinquency on subsequent adolescent delinquency [e.g., (15)], which are also predicted by social learning theories (13, 14). However, what is less clear is whether an *interplay* between such parenting indices and delinquent peer influences could additionally predict adolescent delinquency. Social learning theories do not exclude such an interaction, but these theories do not explicitly postulate such hypotheses either. Although we are not aware of any specific theories that explicitly hypothesize an interaction between parent-adolescent relationship quality and peer delinquency in the prediction of adolescent delinquency, some empirical studies have found support for such a hypothesis.

Namely, a handful of cross-sectional [e.g., (20, 21)] and longitudinal (22, 23) studies that have investigated whether parent-adolescent relationship quality can serve as a moderator between delinquent peer affiliation more generally, and subsequent adolescent delinquency. It should be noted, that conflicting results have been reported however, as some studies found support for this hypothesis (20, 23, 24), whereas others have not (21, 22, 25). Perhaps the general *delinquent peer affiliation* measure that was used in these studies could at least partially explain the contradicting findings, as these studies investigated the effect of affiliation with delinquent peers on subsequent adolescent delinquency, but neglected whether perceived delinquent peer norms or overt peer pressure were present. Thus, the mechanism behind why delinquent peer affiliation might predict adolescent delinquency in the first place, is less clear [for a critical review on this issue see (26)]. Furthermore, presumably, parent-adolescent relationship quality might influence the link between peer delinquency and adolescent delinquency, only in the presence of heightened delinquent peer norms or peer pressure. However, we are not aware of existing studies that have specifically examined whether perceived delinquent peer norms and overt peer pressure predict adolescent delinquency and whether this link is moderated by parent-adolescent relationship quality within the same study sample.

Finally, it is important to further examine whether adolescent phase and gender moderate the above-described hypothesized independent and interdependent/interaction longitudinal links. First, adolescence is a heterogeneous period, with delinquency typically peaking in mid- or late adolescence [ages 15–17 (1, 27, 28)]. When it comes to peer influence, early adolescence has been recently theorized to be most sensitive to peer influence due to neurodevelopmental changes triggered by puberty during this period (29, 30). Namely, *social neurodevelopmental imbalance models* suggest that peers (particularly the *presence* of peers) are socially rewarding during adolescence. Peer presence is theorized to trigger the reward-related brain regions, leading adolescents to place higher value on arousing motivational or rewarding stimuli (29, 30). Hence, perhaps this effect could also explain why adolescents are influenced by peer socialization, and perhaps more so during early adolescence.

Tangential support for the general notion of this social neurodevelopmental imbalance model has been found in some empirical research. For example, there is evidence that peer influence might have stronger effects for early compared to middle adolescence, because there is negligible growth in the capacity to resist peer influence from early to middle adolescence, but this capacity increases throughout middle adolescence (31). Thus middle adolescents are expected to be more resistant to peer influence (31). Similarly, other studies show that peer approval and conformity decrease during middle and late adolescence (32). However, more recent evaluations of associations between pubertal development and risk-taking with respect to social neuro-developmental imbalance models suggest that deviant peer influence might not be stronger during early adolescence, as parents are still relatively vigilant of their young adolescents' behavior (33). Thus, we explore whether peer and parent processes in relation to adolescent delinquency are similar or differ across different developmental phases. As for the parent-adolescent relationship, studies suggest that particularly during early adolescence, conflict between parents and their offsprings increases [for a review: (7)].

Gender effects during adolescence are important to consider too, particularly when investigating adolescent delinquency. First, males outnumber females in delinquency prevalence rates [e.g., (34)] -and perhaps not surprisingly- most research on delinquency is conducted with male participants [cf (22)]. Furthermore, as for peer influences, overall, boys have been shown to be more vulnerable to peer pressure [see e.g., (31, 35)]. However, perhaps unexpectedly, other studies show that females report more peer pressure than males (36). Finally, girls interact and spend more time with their parents compared to boys, thus this should provide more opportunities for girls to be influenced by their parents (21, 37).

The above-described findings could suggest that developmental changes in parent and peer influence across adolescent phase and gender could affect the level of adolescent delinquency over time [cf (38)]. The only study we are aware of that considered both gender and adolescent phase effects in peer and parent influences on adolescent problem behaviors (including delinquency), and thus came close to studying some of the current hypotheses is (38). This study reported that indirect peer pressure^1^ was only predictive of early adolescent boys' delinquency, whereas this was neither the case for middle/late adolescent boys, and nor for girls in any adolescent phase (38). As for parent influences, the study of Worthen (38) found that negativity toward parents did not predict adolescent problem behaviors, for boys or girls, and not in any of the adolescent phases. However, other parenting indices such as parental control were relevant predictors, for boys and/or girls during certain phases of adolescence. Worthen (38) is clearly a valuable and unique comprehensive study. However, it should be noted (38) was based on cross-sectional data, and thus did not control for previous levels of delinquency. Additionally, (38) did not investigate possible interaction effects between parents' and peers' influences. Thus the current longitudinal study that examines hypothesized interactions between parent and peers, across different adolescent phases and gender could further add to the literature in unique ways. As far as we know, there is no single theory on adolescent delinquency that is explicit about combined gender and adolescent phase moderation effects of parent and peer influences. Nevertheless, tangential empirical evidence [e.g., (38)] suggests that such moderation effects are relevant to consider.

Taken together, the current 1-year longitudinal study including two age groups of early and mid-adolescents between the ages 12–15 (*N* = 602) at time point one, investigated parent and peer predictors of minor delinquency (i.e., stealing and vandalism) 1 year later at time point 2. Specifically, extrapolating from social learning theories, such as Coercion Theory, the current study was designed to test whether higher levels of negative mother-adolescent relationship quality moderates the hypothesized links between delinquent peer norms and peer pressure to engage in delinquency and subsequent adolescent delinquency 1 year later, while controlling for delinquency levels in the previous year. Additionally, we explore gender and adolescent phase (early vs. middle adolescence) moderation effects in all of these independent and interaction links.

## Materials and methods

### Participants

Adolescents in the current study were from the first two waves of a larger longitudinal study in the Netherlands on adolescent risk-taking [i.e., (39)]. Data-collections began in 2012, and were conducted 1 year apart. At baseline, 370 (61.6%) adolescents identified as Dutch while the remaining 231 (30.9%) adolescents identified with other ethnic minority groups, and they were from socio-economically heterogeneous families (39). At wave 1 and 2 the sample consisted of 602 (46.42%; *n* = 279 female) and 582 (45.40% female*; n* = 264;) adolescents respectively. At baseline (year 1) adolescents were 13.50 years (*SD* = 1.23) and were in their 1st (42.5% girl; *n* = 124) or 3rd year (50.2% female; *n* = 125) of high school. At baseline, most adolescents in their 1st year of high-school were between the ages 12–13 and most adolescents in their 3rd year of high school were between the ages 14–15. These age periods (i.e., based on high school grade) were used to categorize the adolescents into “early adolescence phase” and “middle adolescence phase,” respectively.

### Procedure

The data-collections took place at schools throughout The Netherlands during regular school hours, and were led by trained research assistants, who were all bachelor and master psychology students. Parents received information letters about the research project as well as dissent letters that could be returned to the schools if they wished to not allow their adolescents to participate. Participants could choose to receive a chocolate candy worth 2 euros as a participation prize, or have their name entered in a raffle for a chance to win a 50 euro gift voucher.

### Materials

*Delinquency* was measured with 7 items, that tapped vandalism (1 item*; Have you ever damaged something on purpose, such as a bus shelter, a window, a car or a seat in the bus or train?)* and property crime (4 items that related to theft) subscales of the International Self-Reported Delinquency questionnaire [ISRD; (40, 41)]. An example of a theft item is “*Have you ever stolen something from a store or warehouse.”* An additional vandalism item “*Have you ever tampered or ruined (vandalize) objects on the streets or inside a building with paint, graffiti, or markers*”? from another delinquency questionnaire was also used [i.e., (42)]. From that same questionnaire, we also included the additional item “*Have you ever done something for which you were arrested by the police?*” (42). The answer-categories for all of the items were: 0 = Never; 0 = Yes, but that was longer than 12 months ago; 1 = Yes, once in the past 12 months; 2 = Yes, twice in the past 12 months; 3 = Yes, three times or more during the past 12 months. For the current study we only focused on delinquency within the last 12 months, thus adolescents who indicated that they have committed a delinquent act in the past, but have not done so in the past 12 months, were coded as 0 and were included in the analyses. All items loaded on one factor (please see Supplemental Materials). An overall mean score was computed of the items, with higher means indicating higher levels of delinquent acts. The Cronbach's alpha's for year 1 and 2 were 0.73 and 0.82, respectively, indicating adequate reliability.

Delinquent Peer Norms in year 1 was measured with the question: How would the majority of your friends react if you would steal something, or buy something that was stolen? The answer categories ranged from “Fully approve it” (=1) to “strongly disapprove it” (=5). We adapted this question from a previous study (i.e., Van Keulen et al. (submitted). Scores were reversed coded for the current analyses, with higher scores denoting higher levels of delinquent peer norms.

*Perceived Peer pressure* in year 1 was measured with two selected items on the Peer Pressure Inventory [PPI; (43)] that concerned stealing and vandalism. Thus we used specifically items that overlapped with the delinquency questionnaire that we administered (see above). For the stealing question, participants had to indicate whether they experienced peer pressure to “*not shoplift or steal anything” vs. “to steal something (shoplift, raid a locker, etc.)*.” For the vandalism question, participants had to indicate whether they experienced peer pressure to “*not trash things or vandalize property” vs. “to trash or vandalize things (write on walls, break windows, etc.)”*. After participants had selected which statement corresponded with their experience, they further had to indicate to what extent that statement is true for them (i.e., “A Little,” “Somewhat” or “A Lot”). However, there was also a “No Pressure” answer option that participants could choose, if they did not experience peer pressure to engage (or not to engage) in the delinquent behaviors. Scores ranged from −3 to 3, with a score of 0 indicating “No peer pressure.” An overall mean score was computed, higher mean scores indicated more peer pressure to engage in delinquent behaviors.

*Negative mother-adolescent relationship quality* was measured with the Negative Interaction scale of the Network of Relationships Inventory [NRI; (44)]. Negative interactions were assessed via conflict (three items; e.g., “How much do you and your mother disagree and quarrel?”) and antagonism (three items; e.g., “How much do you and your mother hassle or nag one another?”) subscales, on a 5-point Likert scale ranging from 1 (*little to none*) to 5 (*could not be more*). All items loaded on one factor (please see Supplemental Materials). A mean score was computed, with higher means indicating higher levels of negative mother-adolescent relationship quality. The Cronbach's alpha was .90, denoting excellent reliability.

### Statistical approach

In Mplus 7.11 (45), we first ran a model (Model A), including multiple path-analyses while controlling for delinquency at T1. Specifically, in model A (non multi-group model) we simultaneously regressed delinquency (T2) on delinquency (T1), perceived peer pressure (T1), delinquent peer norms (T1), mother-adolescent relationship quality (T1), and on the interaction term constituting an interaction between peer norms and mother-adolescent relationship quality (T1), as well as on the interaction term between perceived peer pressure and negative mother-adolescent relationship quality. We mean centered all variables to facilitate the interpretation of the hypothesized interaction effects.

To test for gender and adolescent phase (i.e., early adolescents vs. middle adolescents) moderation effects, we additionally specified a multi-group model (model B). As mentioned above, adolescents in year 1 of high school at baseline were classified as “early adolescents” vs. “middle adolescents” who were in their 3rd year of high-school. Thus, Model B had 4 subgroups, namely: (1) early adolescent girls (*N* = 140), (2) middle adolescent girls (*N* = 191), (3) early adolescent boys (*N* = 185), and (4) middle adolescent boys (*N* = 199). If a significant interaction effect was found for any of these subgroups, we followed up with a Wald test to investigate whether the magnitude of the effect significantly differed across groups.

Considering that our moderator (i.e., negative mother-adolescent relationship quality) is continuous, to probe any significant moderation effects, we used the Johnson-Neyman (J-N) technique that allowed us to plot CI's around simple slopes for all relevant values of the moderator (46–48) and calculate regions of significance. According to this procedure, negative mother-adolescent relationship quality moderates the relationship between the peer factors and delinquency for values of the moderator where the confidence bands do not contain zero. Accordingly, these identified values demarcate the boundaries of significance of the effect of the peer factors (independent variables) on delinquency (dependent variable) along the continuum of the scale for negative mother-adolescent relationship quality (moderator). This designated area(s) is more commonly called the “region of significance.” Thus this procedure differs from the limited “pick a point” procedures in more traditional ANOVA approaches, where researchers investigate a continuous variable, but only test its effect at a few (often arbitrary) values. Instead following the J-N procedure, it is not required to arbitrarily choose a value for the moderator at which the conditional effects of the independent variables are estimated (49).

A Robust Maximum Likelihood estimator (MLR) was used, which accounted for non-normality and ensured that incomplete data could be included in the analyses (50). We ran *Little's MCAR test* (51). At wave 1 (*N* = 602), a maximum of 0.2% of the items were missing for delinquency and for the delinquency norm item, 12.3% of the peer pressure items were missing, and a maximum of 20.3% of the mother-adolescent negative relationship quality items were missing. The mother-adolescent relationship quality items had the most missings because those questions were toward the end of the questionnaire. Due to time constraints at some schools, some participants did not reach the end of the questionnaire. At wave 2 (*N* = 582), a maximum of 1.4 % of the delinquency items were missing. The *Little's MCAR test* across these variables and waves showed that the missings were completely at random (Chi-Square (261) = 240.565, *p* = 0.813). Hence, all missing items were dealt with using the Full Information Maximum Likelihood (FIML) algorithm (45). All the models had a perfect fit to the data (i.e., just-identified). Finally, we used a stringent *p*-value of *p* < 0.01 (instead of the traditional *p* < 0.05)^2^

## Results

### Descriptive statistics

In Table 1 the descriptive statistics can be found, and the correlations between the variables of interest are in Table 2. In year two 9.6% of the adolescents indicated that in the last 12 months, they did something for which they were arrested at least one time by the police. Frequencies on delinquency per item are available in the Supplemental Materials. There were some significant differences between boys and girls on the mean scores of the variables of interest at a *p* < 0.01 level (Table 3). Boys scored significantly higher than girls on delinquency [*t*_(404.51)_ = 4.33, *p* < 0.001], and on delinquent peer norms [*t*_(598.36)_ = 5.43, *p* < 0.001]. However, there were no significant gender differences in delinquency at year 1 [*t*_(596.54)_ = 2.37, *p* = 0.018], for perceived peer pressure [*t*_(528)_ = 1.97, *p* = 0.049] and mother-adolescent negative relationship quality [*t*_(486)_ = −1.72, *p* = 0.086]. As for adolescent phase, no significant differences existed in the mean scores of early vs. middle adolescents at a *p* < 0.01 level (Table 4). *T*-tests information: delinquency T1 [t _(598)_ = −1.74, *p* = 0.082]; delinquency T2 [*t*_(457)_ = −0.88, *p* = 0.381]; peer norms [*t*_(595.20)_ = −1.15, *p* = 0.252]; perceived peer pressure [*t*_(442.80)_ = −2.28, *p* = 0.023]; mother-adolescent negative relationship quality [*t*_(486)_ = −1.427, *p* = 0.154]. All predictor variables were significantly correlated with delinquency in year 1. Furthermore, peer norms and mother-adolescent negative relationship quality were significantly correlated with delinquency in year 2. All correlations were in the expected directions.

**Table 1:** Means and standard deviations of variables of interest.

	**Mean (*SD*)**	**Min, Max**
Delinquency wave 1	0.09 (0.26)	0, 2.71
Delinquency wave 2	0.14 (0.38)	0, 3
Peer norms wave 1	2.08 (1.02)	1, 5
Peer pressure wave 1	−0.56 (1.56)	−3, 3
M-A conflict wave 1	1.77 (0.82)	1, 5

*M-A conflict, negative mother-adolescent relationship quality*.

**Table 2:** Bivariate correlations between variables of interest.

	**1**	**2**	**3**	**4**	**5**
1. Delinquency W1	–				
2. Delinquency W2	0.446^**^	–			
3. Peer pressure W1	0.147^**^	0.059	–		
4. M-A conflict W1	0.216^**^	0.134^**^	0.086	–	
5. Peer norms W1	0.339^**^	0.241^**^	0.153^**^	0.117^**^	–

** *p < 0.01*.

*M-A conflict, Negative mother-adolescent relationship quality; W1, wave 1; W2, Wave 2*.

**Table 3:** Means and SD's per gender.

**Variable**	**Gender**	**Mean**	***SD***
Delinquency T1	Boy	0.12	0.27
	Girl	0.07	0.25
Delinquency T2	Boy	0.20	0.49
	Girl	0.07	0.18
Peer norms	Boy	2.28	1.04
	Girl	1.85	0.94
Peer pressure	Boy	−0.43	1.51
	Girl	−0.070	1.60
M-A conflict	Boy	1.71	0.77
	Girl	1.83	0.87

**Table 4:** Means and SD's per adolescent phase.

**Variable**	**Phase**	**Mean**	***SD***
Delinquency T1	Early	0.07	0.20
	Middle	0.11	0.31
Delinquency T2	Early	0.12	0.29
	Middle	0.15	0.47
Peer norms	Early	2.03	0.94
	Middle	2.13	1.08
Peer pressure	Early	−0.73	1.76
	Middle	−0.42	1.35
M-A conflict	Early	1.71	0.81
	Middle	1.81	0.83

### Main analyses

**Figure 1:**
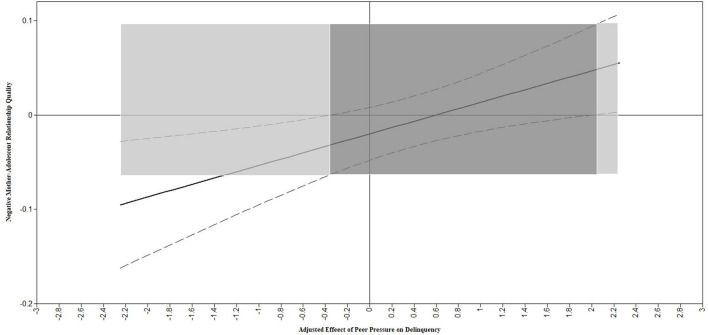
Subgroup: Early adolescent boys. The solid plot line shows that the more negative mother-adolescent relationship quality (i.e., the moderator; x-axis), the more strongly peer pressure to engage in delinquency predicts adolescent delinquency (y-axis = the adjusted effect of peer pressure on delinquency). The dashed curved lines above and below the solid plot line represents 99% confidence bands (upper confidence interval and lower confidence interval, respectively) around the adjusted effect of peer pressure on adolescent delinquency. Accordingly, the dark gray shaded area represents the non-significant values of the moderator (the confidence bands includes the possibility of the adjusted effect of peer pressure on delinquency being equal to 0), and the light gray shaded areas to the left and right represent the regions of significance.

The model without the multi-group comparisons (model A; Table 5) yielded nonsignificant findings, except for a significant effect of delinquency in year 1 on delinquency in year 2. That is, delinquent peer norms, peer pressure, negative mother-adolescent relationship quality in year 1 and the interaction between these peer and parent factors did not predict delinquency in year 2. Next, for the multi-group model (Model B; Table 6), the only significant main effect that existed was for year 1 delinquency in all subgroups except for the early adolescent girls subgroup. Additionally, an interaction effect emerged. Specifically, for early adolescent boys, the link between perceived peer pressure and delinquency was moderated by negative mother-adolescent relationship quality (*b* = 0.03; *p* = 0.006; Johnson-Neyman plots are presented in Figure 1). The plot shows that the more negative mother-adolescent relationship quality in year one, the more strongly delinquent peer pressure positively predicts higher levels of delinquency 1 year later, whereas the lesser negative mother-adolescent relationship quality in year one, the more strongly peer pressure negatively predicts delinquency 1 year later. Specifically, the regions of significance analysis revealed that for early adolescent boys who score 2.05 or above average (*p* = 0.048) on negative mother-adolescent relationship quality, peer pressure to engage in delinquency positively predicts delinquency levels 1 year later. However, adolescents who score 0.35 or below average (*p* = 0.048) on negative mother-adolescent relationship quality, peer pressure negatively predicts delinquency levels 1 year later. Thus, on the one hand there is an *exacerbating effect* of high mother-adolescent negative relationship quality, while on the other hand there is a *buffering/protective* effect of mother-adolescent negative relationship quality on how peer pressure predicts delinquency in early adolescent boys. Follow-up comparisons of the significant interaction in the early adolescent boys subgroup vs. the same interaction in the other three subgroups, showed an overall significant moderation effect of adolescent phase and gender **[**Wald χ2 _(3)_ = 12.81; *p* = 0.005].

**Table 5:** Model A: Non-multi-group model.

	**b**	***SE***	**Beta**	**99%CI of b**	***P*-value**
**PREDICTORS**					
Delinquency (Yr. 1)	0.60^**^	0.15	0.41^**^	0.211, 0.997	<0.001
Negative relationship quality	0.02	0.04	0.04	−0.090, 0.126	0.670
Peer pressure	0.01	0.01	0.03	−0.017, 0.029	0.488
Peer norms	0.03	0.02	0.07	−0.022, 0.076	0.162
Peer norms × negative relationship quality	0.04	0.03	0.13	−0.043, 0.113	0.248
Peer norms × negative relationship quality	−0.07	0.03	−0.16	−0.140, 0.009	0.024

** *p < 0.01*.

**Table 6:** Model B: Muti-group model.

**Predictors**	***b***	***SE***	**Beta**	**99%CI of b**	***P-*value**
**EARLY ADOLESCENT GIRLS**					
Delinquency (Yr. 1)	0.17	0.14	0.16	−0.201, 0.544	0.235
Negative relationship quality	0.02	0.04	0.11	−0.071, 0.113	0.553
Peer pressure	−0.00	0.01	−0.01	−0.032, 0.031	0.963
Peer norms	0.08	0.03	0.41	−0.005, 0.170	0.015
Peer pressure × negative relationship quality	−0.03	0.04	−0.28	−0.124, 0.062	0.397
Peer norms × negative relationship quality	−0.03	0.09	−0.11	−0.251, 0.19	0.743
**MIDDLE ADOLESCENT GIRLS**					
Delinquency (Yr. 1)	0.36^**^	0.08	0.59	0.168, 0.553	<0.001
Negative relationship quality	0.05	0.02	0.22	−0.013, 0.107	0.042
Peer pressure	−0.01	0.01	−0.11	−0.048, 0.020	0.292
Peer norms	0.01	0.01	0.03	−0.030, 0.041	0.695
Peer pressure × negative relationship quality	−0.03	0.02	−0.23	−0.094, 0.032	0.207
Peer norms × negative relationship quality	0.01	0.02	0.04	−0.031, 0.045	0.621
**EARLY ADOLESCENT BOYS**					
Delinquency (Yr. 1)	0.45^**^	0.17	0.30	0.020, 0.883	0.007
Negative relationship quality	−0.02	0.04	−0.05	−0.133, 0.088	0.597
Peer pressure	−0.02	0.01	−0.10	−0.057, 0.017	0.158
Peer norms	0.03	0.03	0.08	−0.050, 0.108	0.348
Peer pressure × negative relationship quality	0.03^**^	0.01	0.14	0.002, 0.064	0.006
Peer norms × negative relationship quality	−0.07	0.04	−0.19	−0.184, 0.045	0.119
**MIDDLE ADOLESCENT BOYS**					
Delinquency (Yr. 1)	0.73^**^	0.27	0.38	0.031, 1.423	0.007
Negative relationship quality	−0.05	0.09	−0.06	−0.273, 0.184	0.615
Peer pressure	0.07	0.03	0.16	−0.004, 0.146	0.014
Peer Norms	−0.01	0.04	−0.03	−0.124, 0.095	0.737
Peer pressure × negative relationship quality	0.19	0.07	0.44	−0.001, 0.381	0.010
Peer norms × negative relationship quality	−0.15	0.07	−0.21	−0.318, 0.027	0.030

** *p < 0.01*.

## Discussion

The current longitudinal study investigated a possible interplay between parent and peer factors in adolescent delinquency. Extrapolating from social learning theories such as Coercion theory, we investigated whether negative mother-adolescent relationship quality moderates the hypothesized effects of delinquent peer norms and peer pressure on adolescent delinquency 1 year later and whether gender and adolescent phase (12–13 years vs. 14–15 years) moderate these linkages. Our main analyses without the adolescent phase by gender multi-group models only yielded significant results for prior adolescent delinquency. That is, higher levels of delinquency in 12–15 year old boys and girls predicted higher levels of delinquency 1 year later, however there were no significant effects for delinquent peer norms, perceived peer pressure, negative mother-adolescent relationship quality, or for the interaction between these peer and parent factors.

When looking specifically at early adolescents, results showed that for early adolescent boys, lower than average mother-son negative relationship quality (i.e., conflict and antagonism) attenuated the relationship between delinquent peer pressure and adolescent delinquency. Conversely, higher than average mother-son relationship quality exacerbated the link between of delinquent peer pressure and delinquency. In other words, the lesser the negative mother-adolescent relationship quality, the less delinquent behavior adolescents will portray when confronted with increased delinquent peer pressure. However, the higher negative mother-adolescent relationship quality, the more delinquency early adolescent boys will portray when faced with increased delinquent peer pressure. Thus for early adolescent boys, low mother-adolescent negative relationship quality acts as a protective factor against the effect of peer pressure on adolescent delinquency, whereas high mother-adolescent negative relationship quality acts as a risk factor. Finally, we did not find any significant interaction effects for middle-adolescents, or early adolescent girls, however.

Coercion theory hypothesizes that children/adolescents who have negative interactions with their parents are more likely to subsequently associate with delinquent peers (13, 14); see also: (5). Such delinquent peer affiliations would typically be characterized by delinquent peer norms and peer pressure. On the one hand, our results suggest that high negative mother-adolescent relationship quality further amplifies the adverse consequences of delinquent peer influences (i.e., delinquent peer pressure) on early adolescent boys delinquency. On the other hand, in the event of delinquent peer pressure, lower levels of mother-adolescent negative relationship quality can reverse the effect that such delinquent peer influences can have on adolescent own delinquency. Thus it is hopeful news that when early adolescent boys—that have low levels of negative relationship quality with their mothers—are faced with delinquent peer pressure, they appear to be more capable of resisting and even undoing the amplifying effect of delinquent peer pressure on their delinquency. It is important to consider that results of studies on such interaction effects of parenting and delinquent peers on adolescent delinquency have been mixed, however. Nevertheless, one study that somewhat mirrors our results showed that positive mother-adolescent relationship quality when adolescents were in the 7th or 8th grade (ages 12–14; mean age 13.4) attenuated the link from peer problem behaviors to adolescent problem behaviors such as delinquency 1 year later [(24); see also (23)]. However, contrary to the current results, (24) additionally found a main effect of peer delinquency. Finally, also noteworthy is that in addition to the above-described interaction model, (24) tested a mediational model wherein mother-adolescent relationship quality was hypothesized to predict peer delinquency which in return predicted early adolescent delinquency (these cascading links are consistent with Coercion theory). Additionally, a cumulative model was also tested in (24), which included a cumulative index of these peer and parent predictors. Interestingly, only the interaction model with parent and peer factors predicted adolescent delinquency (24), which provides further support for the interaction effect that was found in the current study. However, potential moderational effects of adolescent phase was not taken into account in Mason et al. (24), limiting the comparisons that can be made with the current study. As for gender, (24) did report that attachment to mothers was significantly more strongly correlated with problem behaviors in early adolescent *boys* compared to early adolescent girls. This finding of gender differences in Mason et al. (24) mirrors the gender by adolescent phase moderation effect that was found in the current study. That is, mother-adolescent relationship quality is only relevant for (early-) adolescent boys' delinquency.

To this end, the current results raise three questions in particular. First why are peer and parent influences only relevant for adolescent delinquency during *early* adolescence but not in middle-adolescence? Secondly, why are peer and parent influences only relevant for adolescent delinquency for *boys*, but not for girls? Finally, why were peer norms not found to be a relevant predictor for any of the gender by adolescent phase subgroups?

To answer the first question, coercion theory is not explicit about whether parent and peer effects will differ across the adolescent phases. Nevertheless, our results support the general hypothesis of many social learning theories that delinquent peer socialization predicts adolescent delinquency, but at the same time the current results suggest that this occurrence only predicts delinquency in *early* adolescence. Thus, generally, the current results are in line with some empirical evidence that imply that particularly early adolescents (compared to older adolescence) are more vulnerable to delinquent peers, perhaps because early adolescents have not yet fully developed the capacity to resist peer influence [(31); see also (32)]. As for parent influence during *early* adolescence, our results are in line with developmental theorists that hypothesize that parents could continue to exert influence on their adolescents, despite the growing influence of peers [for a review see: (7)]. During middle adolescence, we did not find that the relationship quality between adolescents and their parents was significant, however. This is perhaps because the parent-child bond decreases during middle adolescence, which could suggest that parents could become less influential during this adolescent phase (54).

Revisiting the second question, coercion theory does not explicitly delineate differences in gender. Nevertheless, our finding that perceived peer pressure is more relevant for early adolescent *boys'* delinquency, is in line with studies that show that boys are more vulnerable to peer pressure (31, 35). As for parenting, we did not find any significant effects for girls, perhaps because mother-daughter negative interactions (compared to mother-son negative interactions) might be a normative occurrence. Alternatively, another aspect of parenting might be more relevant for predicting girls' delinquency. For example, parents tend to monitor and supervise their early adolescent daughters more than they do with their early adolescent sons [see e.g., (38)]. Thus this could affect the type of peers girls socialize with, which could in turn affect the levels of early adolescent girls' delinquency (21, 37, 38).

To address the third and final question, perhaps perceived peer pressure is a relevant predictor vs. peer norms, simply because it is a form of *direct* peer influence, although empirical evidence on adolescent substance use suggest peer norms might be more influential (9). We investigated peer norms and peer pressure simultaneously in the same models. Thus perhaps our results could suggest that even though peer norms are salient, if peer pressure exists, it might override any influence that peer norms might have, particularly so for early adolescent boys. We are not aware of any studies that have explicitly investigated this, however. These suggestions are pure speculations, and thus need to be empirically studied before firmer conclusions can be drawn. An alternative explanation is that our peer norms measure was not sensitive enough, as it only tapped stealing and buying things that were stolen, whereas our delinquency outcome measure was more diverse. Thus perhaps a more diverse measure of peer norms would have been more suitable.

Taken together, our results are not fully consistent with existing theories, particularly because we found differential effects across gender and adolescent phase, while most theories are not so specific about such moderation effects. Nevertheless, as mentioned earlier, we know of at least one study that considered both gender and adolescent phase effects in peer and parent influences on adolescent delinquency (38). Consistent with the current findings, using a cross-sectional samples, Worthen (38) also reported that peer pressure was only predictive of early adolescent boys' delinquency, whereas this was neither the case for middle/late adolescent boys, and nor for girls in any adolescent phase. Additionally, as for parental influences, negativity toward parents was not a significant independent predictor of delinquency in any phase of adolescence for both boys and girls, which is consistent with the current findings. Worthen (38) showed that other parenting aspects could still have a main effect on adolescent delinquency though. For example, parental monitoring was shown to predict delinquency for middle adolescents, although this was only found to be the case for boys. Worthen (38), did not investigate interaction effects between peer and parent factors, however, and therefore its results are not directly comparable with the current results. Taken together, both (38), and the above-described (24) reported findings that are similar to the current findings. However, the methodologies and designs used in those studies limit the comparisons that can be made with the current study. Nevertheless, Mason et al. (24) and (38) provide tangential evidence that our results could imply that early adolescent boys whose relationships with their mothers are characterized by low levels of conflict and antagonism are able to stave off perceived peer pressure.

To summarize, the results of the current study suggest that a prominent aspect of Patterson's coercion theory about the adverse effect of parent-adolescent negative relationship quality on adolescent delinquency is most meaningful for adolescents who are pressured by their friends to engage in delinquency, or perceive this to be the case. However, we found that both *low* and high levels of mother-adolescent negative relationship quality could moderate the links between delinquent peer factors and adolescent delinquency. Importantly, we found that this interplay between mothers and peer factors is only present in *early adolescent boys*. Our differential findings for boys and girls and for early vs. middle adolescence complicate the fundamental premises of social learning theories that suggest that mere delinquent peer affiliation is a predictor of adolescent delinquency, as our results show that this might be particularly true for boys, and for early adolescence (compared to middle adolescence). Such moderation effects were perhaps masked in prior studies because the assumed specific peer influence aspects that link peer delinquency to higher levels of adolescent delinquency were not assessed. To conclude, the present results propose that social learning theories on peer influences in delinquency would likely benefit from being more refined, by taking developmental and gender differences into account, but also by being more specific about the aspects of delinquent peer influence that predict adolescent delinquency. Furthermore, acknowledging that such differential peer factors might also be interconnected with factors outside of the peer context (e.g., the family context) could also advance our understanding of how such complex peer influences operate.

As scholars have already noted, peer influence processes are complex and wide-ranging, accordingly, a comprehensive framework for peer influences is crucially needed in order to reconcile findings across the existing various methodologically diverse studies with different designs and sample characteristics [for a critical review see: (9, 55). Nevertheless, the current study has pinpointed that during *early* adolescence, direct/overt forms of peer pressure (which is moderated by mother-adolescent relationship quality) might be more relevant for early adolescent boys, but not for mid-adolescent boys, or for early-mid-adolescent girls. *Why* this is the case, and *if* these gender and adolescent phase differences in such peer influences can be replicated in other samples await future research.

### Strengths, limitations, and future directions

The current longitudinal study could provide some new insights into possible components of delinquent peer socialization that predict subsequent adolescent delinquency, albeit the significant results we found are only relevant particularly for early adolescent boys. Of note is that capitalizing on a short-term longitudinal design, we highlighted a potential prevention component for adolescent delinquency. That is, our time-lagged interaction assessed 1 year earlier suggests that mother-adolescent relationship quality at an earlier point in time can be an influential factor in determining whether delinquent peer pressure will lead to an increase in adolescent delinquency in the future. However, despite these strengths, there are also some limitations that need to be addressed. Of note is that despite that nearly 10% of the adolescents (which is a substantial amount) in year 2 reported being arrested at least one time by the police for delinquent behavior, the overall level of delinquency was on the lower side in the current sample. This is perhaps because we did not assess violent delinquent behavior (e.g., fighting). Nevertheless, it would be interesting to investigate whether the current results would replicate in an at-risk sample, or a clinical sample with higher levels of delinquency. Readers should also be aware that possibly boys vs. girls across the different adolescent phases could have interpreted the delinquency items differently, which might have influenced the gender and adolescent phase differences we found. A limitation concerning our measures is that our peer norm measure only tapped stealing, whereas it should have preferably tapped more types of delinquency.

Next, although we examined effects from multiple layers of adolescents' social network (parents and friends), we did not consider father and sibling factors, but these significant others are likely also interconnected with adolescents' peer context also. We expect that fathers might have similar effects as mothers whereas siblings might have similar effects as friends [see e.g., (15)]. It should also be noted that in the current study we emphasized the potential negative effects of peers on adolescents' behavior, but peers can also have positive influences [see e.g., (56)]. Similarly, positive aspects of parenting could also be important to consider, but we chose to focus on the negative aspects of parent support, as such parenting indices were shown to be the strongest predictors of adolescent delinquency in a meta-analysis on the relationship between parenting and adolescent delinquency (8). This meta-analytic finding is also in line with the Coercion theory, which is one of the theoretical frameworks of the current paper. We also recommend future studies to investigate coercion theory on a micro-level, and preferably with analyses that allow the testing of bi-directional effects, as this would allow for a more comprehensive test of the theory. As for bi-directional effects, not only might parents influence their children, but children could also influence the way their parents treat them (57, 58).

On a side note, it is relevant to mention that although coercion theory is an influential theory on externalizing problem behavior development, it has also been criticized. Namely, coercion theory claims that it is parental behaviors that causes their offsprings to act in similar ways to their parents. In other words, adolescents learn behaviors from their parents. However, parents and their offsprings might act in similar ways because of genetic confounding [for a discussion see (59)], an important factor which we did not account for in the current study. In fact genetic factors might also influence what type of peers adolescent choose to socialize with [for a critical review on this topic see: (59, 60); see also: (61)]. A related point is that although the peer predictors we investigated might give us more information on delinquent peer influence processes compared to the more traditional method of assessing whether or not friends/peers' delinquency predict adolescents' own delinquency, we did not account for peer selection effects. An experimental design is needed in order to draw firmer conclusions about the specific aspects of peer influence (particularly delinquent peer pressure) that the current study has put forward as possible explanations for the link between peer delinquency and adolescent delinquency.

## Conclusion

The current study showed that when investigating adolescent delinquency, interactions between peer and parent predictors as well as moderation by adolescent phase and gender are important to consider. Namely, particularly for early adolescent boys, we found that influences of delinquent peers and parents do not operate independently, as it is the interplay between peer pressure to engage in delinquency and high negative mother-adolescent relationship quality that predicted higher levels of delinquency. However, importantly, the reverse is also true. That is, low levels of negative relationship quality between mothers and their early adolescent sons, can minimize the amplifying effect that deviant peers have on adolescent delinquency. Thus the current findings have highlighted potentially amendable characteristics of parent-adolescent relationship quality that could possibly make early adolescent boys less vulnerable to delinquent peer pressure, and thus this could be valuable findings for interventions. Accordingly, the findings could perhaps be valuable for interventions if considered within the broader context of what is known on relative parent and peer influences on adolescent delinquency. For example, the results suggest that delinquent peers increase adolescent delinquency, but only under certain conditions, such as when there are higher levels of negative mother-adolescent relationship quality in combination with higher levels of peer pressure to engage in delinquency, and this is only the case for early adolescent boys. Having a healthy relationship with mothers could then possibly break the vicious cycle of the effect of delinquent peer affiliation on delinquency. Namely, adolescents who engage in delinquency typically have delinquent friends who might pressure them to (further) engage in delinquent behaviors. However, it is likely not feasible to include adolescent's entire delinquent peer group in therapy sessions. Hence in such a case, interventions could focus on improving the relationship quality between mothers and their adolescent offsprings, and this could minimize the amplifying effect that delinquent peer pressure has on adolescent delinquency. One of such notable interventions that has proven to be effective for Dutch families [see e.g., (62)] is the Triple P–Positive Parenting Program (63, 64). Inspired by social learning theories, Tripple P is an evidenced-based program that focuses on building positive parent-child interactions and thereby minimizing conduct problems in children and (early) adolescents. The effectiveness of Tripple P has been replicated in many countries (65–67). Another internationally well-established intervention that also targets the parent-child relationship to reduce conduct problems in children and early adolescence is the Incredible Years Program (68, 69).

Considered together, the longitudinal nature of our results suggest that ensuring fewer negative interactions between mothers and adolescents at an earlier time point (in advance) could potentially curtail the adverse effects delinquent peer pressure has on early adolescent boys' delinquency in the future. Thus, the current findings could also provide useful implications for *prevention* efforts in addition to the above-described intervention efforts.

## Ethics statement

This study was carried out in accordance with the ethical standards that were in place at the Faculty of Social and Behavioral Sciences, Utrecht University at the time the study began. Standardized questionnaires and computer tasks were used that were not deemed likely to yield physical or emotional risks for the participants and therefore review by the ethical committee was not deemed necessary and therefore no formal approval was given. All subjects' parents gave passive informed consent for their adolescent's participation in the study and participants could withdraw their participation at any time.

## Author contributions

ID developed the study concept and design, and JD and MvA gave advice and feedback. ID oversaw the data-collection. ID performed the data-analysis and interpretation. ID drafted the manuscript, and JD and MvA provided critical revisions.

### Conflict of interest statement

The authors declare that the research was conducted in the absence of any commercial or financial relationships that could be construed as a potential conflict of interest.

## Footnotes

^1^An example item is: “I sometimes do things because my friends are doing them.”

^2^Please note that the p-value's for the b-values and the beta's slightly differed from each other for some analyses. As recommended by Muthén (52, 53) we are reporting and interpreting significant b-values (52, 53).
