# Supplementary material for: The Relative Roles of Peer and Parent Predictors in Minor Adolescent Delinquency: Exploring Gender and Adolescent Phase Differences
Source: Front Public Health. 2018 Sep 13;6:242. doi: 10.3389/fpubh.2018.00242 (PMC6157422; doi:10.3389/fpubh.2018.00242)
Supplement: Supplementary file 1 [file Table_1.docx]

| Table 1. Frequencies for the delinquency items. | Year 1  % | Year 2  % |
| --- | --- | --- |
| **Have you ever done something for which you were arrested by the police?** |  |  |
| a. Yes, but that was longer than 12 months ago | 91 | 90.4 |
| b. Yes, once in the past 12 months | 6 | 4.7 |
| c. Yes, twice in the past 12 months | 1.3 | 1.7 |
| d. Yes, three times or more during the past 12 months. | 1.7 | 3.1 |
| **Have you ever deliberately (on purpose) destroyed/vandalized something, for example a bus stop, a window, a seating in a tram/train/bus, or a car?** |  |  |
| a. Yes, but that was longer than 12 months ago | 92.2 | 91 |
| b. Yes, once in the past 12 months | 4.5 | 4.9 |
| c. Yes, twice in the past 12 months | 2 | 1.4 |
| d. Yes, three times or more during the past 12 months. | 1.3 | 2.8 |
| **Have you ever tampered or ruined (vandalize) objects on the streets or inside a building with paint, graffiti, or markers?** |  |  |
| a. Yes, but that was longer than 12 months ago | 88.9 | 85.2 |
| b. Yes, once in the past 12 months | 5.2 | 6.3 |
| c. Yes, twice in the past 12 months | 1.8 | 2.8 |
| d. Yes, three times or more during the past 12 months. | 4.2 | 5.7 |
| **Have you ever stolen something from a store or warehouse?** |  |  |
| a. Yes, but that was longer than 12 months ago | 96.5 | 94.8 |
| b. Yes, once in the past 12 months | 1.7 | 1.7 |
| c. Yes, twice in the past 12 months | 0.7 | 1.0 |
| d. Yes, three times or more during the past 12 months. | 1.2 | 2.4 |
| **Have you ever stolen a bicycle, scooter, or motorbike?** |  |  |
| a. Yes, but that was longer than 12 months ago | 98.5 | 97 |
| b. Yes, once in the past 12 months | 0.8 | 0.9 |
| c. Yes, twice in the past 12 months | 0.7 | 0.7 |
| d. Yes, three times or more during the past 12 months. | 0 | 1.4 |
| **Have you ever stolen someone else’s wallet, bag or a different object?** |  |  |
| a. Yes, but that was longer than 12 months ago | 98.8 | 98.4 |
| b. Yes, once in the past 12 months | 0.7 | 0.3 |
| c. Yes, twice in the past 12 months | 0.2 | 0.3 |
| d. Yes, three times or more during the past 12 months. | 0.3 | 0.9 |
| **Have you ever bought or sold something from which you knew or had the feeling that it was stolen?** |  |  |
| a. Yes, but that was longer than 12 months ago | 95.8 | 93.9 |
| b. Yes, once in the past 12 months | 2.5 | 2.1 |
| c. Yes, twice in the past 12 months | 0.5 | 0.9 |
| d. Yes, but that was longer than 12 months ago | 1.2 | 3.1 |
