# Supplementary material for: The Relative Roles of Peer and Parent Predictors in Minor Adolescent Delinquency: Exploring Gender and Adolescent Phase Differences
Source: Front Public Health. 2018 Sep 13;6:242. doi: 10.3389/fpubh.2018.00242 (PMC6157422; doi:10.3389/fpubh.2018.00242)
Supplement: Supplementary file 2 [file Table_2.docx]

**Factor analysis for the Delinquency and NRI scales**

We conducted a principal component analysis (PCA) with a varimax rotation for the delinquency scales at wave 1 (control variable) and delinquency at wave 2 (dependent variable) and on the NRI scale at wave 1 (independent variable; moderator), in order to test the appropriateness of the use of mean scores for these scales.

For the delinquency scale at wave 1, The Kaiser–Meyer–Olkin measure (KMO = .82) was great (Field, 2009), and all KMO values for individual items (i.e., measures of sampling adequacy) were above the acceptable limit of .5 (Field, 2009). Bartlett’s test of sphericity *χ²* (21) = 959.281, *p* < .001, showed that correlations between the items were large enough for a PCA. Initially we ran an explanatory analysis to specify the eigenvalues for each component in the data. Although two components were extracted, strictly speaking, only component 1 met the Kaiser’s criterion of > 1. Component 1 had an initial eigenvalue of 3.004 and component 2 had an initial eigenvalue of .1.008. Thus we re-ran the analysis, while explicitly specifying to extract only one factor. Table 2a shows the loadings for this factor, all factor loadings were >.30, thus all items loaded sufficiently loaded only on one factor. Moreover, the Internal reliability of these items was adequate (Cronbach’s alpha = .73). Thus we decided only to retain this sole factor.

The delinquency scale at wave 2 and the NRI scale at wave 1 had a more unambiguous factor structure. Namely, only 1 component was extracted per scale. For the delinquency scale at wave 2, The Kaiser–Meyer–Olkin measure (KMO = .85) was “great” (Field, 2009), and all KMO values for individual items were above the acceptable limit of .5 (Field, 2009). Bartlett’s test of sphericity *χ²* (21) = 1607.695, *p* < .001 was also adequate. The factor loadings which were all >.30 indicate that all items loaded sufficiently only on one factor, please see Table 2b. For the NRI, The Kaiser–Meyer–Olkin measure (KMO = .906) was “great” (Field, 2009), and all KMO values for individual items were above the acceptable limit of .5 (Field, 2009). Bartlett’s test of sphericity *χ²* (15) = 1947.271, *p* < .001 was also adequate. The factor-loadings were all >.30 suggesting that all items loaded sufficiently on one factor (Table 2c).

Table 2a.

Factor Loadings for the Delinquency Scale at T1

| **Variable** | **Factor loading** | |
| --- | --- | --- |
| Have you ever stolen someone else’s wallet, bag or a different object? | | .739 |
| Have you ever stolen something from a store or warehouse? | | .728 |
| Have you ever done something for which you were arrested by the police? | | .671 |
| Have you ever stolen someone else’s wallet, bag or a different object? | | .637 |
| Have you ever bought or sold something from which you knew or had the feeling that it was stolen? | | .635 |
| Have you ever deliberately (on purpose) destroyed/vandalized something, for example a bus stop, a window, a seating in a tram/train/bus, or a car | | .619 |
| Have you ever tampered or ruined (vandalize) objects on the streets or inside a building with paint, graffiti, or markers? | | .569 |

Table 2b

Factor Loadings for the Delinquency Scale at T2

**Factor**

| **Variable** | **Loading** | |
| --- | --- | --- |
| Have you ever done something for which you were arrested by the police? | | .725 |
| Have you ever deliberately (on purpose) destroyed/vandalized something, for example a bus stop, a window, a seating in a tram/train/bus, or a car? | | .743 |
| Have you ever tampered or ruined (vandalize) objects on the streets or inside a building with paint, graffiti, or markers? | | .498 |
| Have you ever stolen something from a store or warehouse? | | .748 |
| Have you ever stolen a bicycle, scooter, or motorbike? | | .794 |
| Have you ever stolen someone else’s wallet, bag or a different object? | | .796 |
| Have you ever bought or sold something from which you knew or had the feeling that it was stolen? | | .780 |

Table 2c

Factor Loadings for the NRI Scale

| **Variable** | **Factor loading** |
| --- | --- |
| How often do you and your mother point out each others’ faults or put each other down? | .630 |
| How much do you and your mother say mean or harsh things to each other? | .849 |
| How much do you and your mother get on each other’s nerves? | .871 |
| How often do you and your mother disagree and quarrel? | .871 |
| How much do you and your mother get annoyed with each other’s behavior? | .895 |
| How much do you and your mother hassle or nag one another? | .862 |

**References**

Field, A. *Discovering statistics using SPSS: (and sex and drugs and rock 'n' roll).*

*Introducing Statistical Methods*. Thousand Oaks, CA: Sage Publications (2009).
